# Cost-effectiveness analysis of combined cognitive and vocational rehabilitation in patients with mild-to-moderate TBI: results from a randomized controlled trial

**DOI:** 10.1186/s12913-022-07585-3

**Published:** 2022-02-12

**Authors:** Emilie Isager Howe, Nada Andelic, Silje C R Fure, Cecilie Røe, Helene L Søberg, Torgeir Hellstrøm, Øystein Spjelkavik, Heidi Enehaug, Juan Lu, Helene Ugelstad, Marianne Løvstad, Eline Aas

**Affiliations:** 1 Department of Physical Medicine and Rehabilitation, Oslo University Hospital, Oslo, Norway; 2 Institute of Clinical Medicine, Faculty of Medicine, University of Oslo, Oslo, Norway; 3 Center for Habilitation and Rehabilitation Models and Services (CHARM), Institute of Health and Society, Faculty of Medicine, University of Oslo, Oslo, Norway; 4 Faculty of Health Sciences, Oslo Metropolitan University, Oslo, Norway; 5 The Work Research Institute, Oslo Metropolitan University, Oslo, Norway; 6 Department of Family Medicine and Population Health, Division of Epidemiology, Virginia Commonwealth University, Richmond, USA; 7 Department of Vocational Rehabilitation, Norwegian Labour and Welfare Administration, Oslo, Norway; 8 Department of Research, Sunnaas Rehabilitation Hospital, Nesoddtangen, Norway; 9 Department of Psychology, Faculty of Social Sciences, University of Oslo, Oslo, Norway; 10 Department of Health Management and Health Economics, Institute of Health and Society, Faculty of Medicine, University of Oslo, Oslo, Norway; 11 Health Services Research Unit, Akershus University Hospital, Lørenskog, Norway

**Keywords:** Traumatic brain injury, Health economics, Clinical trial, Vocational rehabilitation

## Abstract

**Background:**

Traumatic brain injury (TBI) represents a financial burden to the healthcare system, patients, their families and society. Rehabilitation interventions with the potential for reducing costs associated with TBI are demanded. This study evaluated the cost-effectiveness of a randomized, controlled, parallel group trial that compared the effectiveness of a combined cognitive and vocational intervention to treatment as usual (TAU) on vocational outcomes.

**Methods:**

One-hundred sixteen participants with mild-to-moderate TBI were recruited from an outpatient clinic at Oslo University Hospital, Norway. They were randomized to a cognitive rehabilitation intervention (Compensatory Cognitive Training, CCT) and Supported Employment (SE) or TAU in a 1:1 ratio. Costs of CCT-SE and TAU, healthcare services, informal care and productivity loss were assessed 3, 6 and 12 months after study inclusion. Cost-effectiveness was evaluated from the difference in number of days until return to pre-injury work levels between CCT-SE and TAU and quality-adjusted life years (QALYs) derived from the EQ-5D-5L across 12 months follow-up. Cost-utility was expressed in incremental cost-effectiveness ratio (ICER).

**Results:**

The mean total costs of healthcare services was € 3,281 in the CCT-SE group and € 2,300 in TAU, informal care was € 2,761 in CCT-SE and € 3,591 in TAU, and productivity loss was € 30,738 in CCT-SE and € 33,401 in TAU. Costs related to productivity loss accounted for 84% of the total costs. From a healthcare perspective, the ICER was € 56 per day earlier back to work in the CCT-SE group. Given a threshold of € 27,500 per QALY gained, adjusting for baseline difference in EQ-5D-5L index values revealed a net monetary benefit (NMB) of € -561 (0.009*27,500–979) from the healthcare perspective, indicating higher incremental costs for the CCT-SE group. From the societal perspective, the NMB was € 1,566 (0.009*27,500-(-1,319)), indicating that the CCT-SE intervention was a cost-effective alternative to TAU.

**Conclusions:**

Costs associated with productivity loss accounted for the majority of costs in both groups and were lower in the CCT-SE group. The CCT-SE intervention was a cost-effective alternative to TAU when considering the societal perspective, but not from a healthcare perspective.

**Trial registration:**

ClinicalTrails.gov NCT03092713.

**Supplementary Information:**

The online version contains supplementary material available at 10.1186/s12913-022-07585-3.

## Background

It is widely recognized that traumatic brain injury (TBI) represents a substantial economic burden to the healthcare system, in addition to the patients, their families and society [1]. The total annual global burden of TBI is estimated to US$ 400 billion [2]. Hence, rehabilitation services with the potential for reducing this burden are needed. Return to work (RTW) is considered an important indicator of recovery and a key element in successful rehabilitation following TBI. However, randomised controlled trials (RCTs) assessing the effectiveness of healthcare services and vocational rehabilitation delivery after TBI are sparse. There is some evidence to suggest that rehabilitation interventions such as multidisciplinary inpatient- and home-based rehabilitation, cognitive-behavioural therapy, cognitive rehabilitation with particular emphasis on compensatory strategies, and work-directed interventions in combination with education/coaching are likely to produce improved care efficiency and RTW, in addition to substantial cost savings [3–6]. Previous studies have demonstrated that any upfront investment in early specialist rehabilitation services are rapidly offset by the cost savings made through increased and faster functional improvements [6, 7]. In addition, a prospective RCT found that a specialist multidisciplinary domiciliary outreach team showed increased independence and fewer care needs, suggesting that specialist community-based rehabilitation in the late phase after TBI also resulted in significant long-term cost savings [8]. However, in a recent study from the UK, group-based memory rehabilitation with 10 weekly sessions of a manualised memory rehabilitation programme did not demonstrate cost-effectives for people with TBI living in the community [9].

We recently published results from a RCT which evaluated whether a combined cognitive (Compensatory Cognitive Training, CCT) and vocational (Supported employment, SE) intervention initiated in the early phase (8–12 weeks following mild-to-moderate TBI) improved work-related outcomes compared to treatment as usual (TAU) [10, 11]. We noted that a significantly larger proportion of patients who received the combined cognitive and vocational intervention had returned to stable employment at 3-month follow-up (81 vs 60%). There were no statistically significant between-group differences in RTW proportion at the 12-month follow-up. However, we noted a difference in median number of days until reaching pre-injury level of employment, with 50% of participants receiving the combined intervention having returned to pre-injury employment levels at 365 days after injury, while the same was true after 415 days for the TAU group. We concluded that the CCT-SE intervention might assist patients with mild-to-moderate TBI, who are still on sick leave 8–12 weeks after injury, in returning earlier to stable employment and preinjury work levels. Nevertheless, to our knowledge, no cost-effectiveness analysis has evaluated CCT-SE or similar interventions following mild-to-moderate TBI. It is difficult to determine the optimal allocation of health care budgets without considering utilization and cost data. Therefore, economic evaluations provide an opportunity to identify optimal strategies for clinical management of persons with mild-to-moderate TBI.

The purpose of this study was thus to describe health care utilization, informal care, production loss and the total costs of providing a combined cognitive and vocational rehabilitation compared to a TAU control group during the intervention period (baseline to month 3 and baseline to month 6) and 12 months after inclusion (baseline to month 12). In addition, we estimated the cost-effectiveness of the combined rehabilitation intervention on mild-to-moderate TBI in terms of difference in number of days until reaching pre-injury work levels and quality-adjusted life-years (QALYs). We hypothesized that the early initiation of a combined rehabilitation intervention would be a more cost-effective alternative to TAU.

## Methods

### Study design and participants

This study is a cost-effectiveness analysis of a randomized, controlled, parallel-group trial that compared a combined cognitive and vocational intervention to multidisciplinary TAU on employment and other clinical outcomes after mild and moderate TBI. Details of the study design, recruitment, randomisation, blinding and data collection are provided in the study protocol [12]. Briefly, the trial was conducted at a specialized TBI outpatient clinic at Oslo university hospital (OUH), Norway, where patients were randomised to a group-based compensatory cognitive training intervention and individualized supported employment (CCT-SE) or individualized outpatient treatment provided by a multidisciplinary team (TAU) in a 1:1 ratio. Participants were recruited and started treatment 8–12 weeks after sustaining a mild or moderate TBI and received the intervention or control group treatment for a total of 6 months.

The trial was performed in accordance with the Declaration of Helsinki, and registered at ClinicalTrials.gov on 28.03.2017 (#NCT03092713). One-hundred and sixteen individuals aged 18–60 years were recruited to the RCT between July 2017 and April 2019. Two participants dropped out from the CCT-SE group and 1 from the TAU group after randomization. See Additional file 1 for flow chart of participant recruitment. Participants had sustained a mild or moderate TBI assessed by a Glasgow Coma Scale (GCS) score of 10–15, loss of consciousness for < 24 h and post-traumatic amnesia for < 7 days. Criteria from the American Congress of Rehabilitation Medicine were used to establish the presence of mild TBI [13]. All participants were employed in a minimum 50% position at the time of injury, but sick listed 50% or more due to post-concussive symptoms assessed by the Rivermead Post Concussion Symptoms Questionnaire [14] 2–3 months post injury. Table 1 provides participants’ demographic, injury- and work-related characteristics at baseline, in addition to EQ-5D-5L index values. There were no statistically significant between group differences on baseline characteristics, with the exception of EQ-5D-5L index values (*p* = 0.013). See Additional file 2 for proportion of responses by level of severity for EQ-5D-5L dimensions at baseline per treatment group.

**Table 1:** Baseline characteristics of participants in the treatment groups

	**CCT-SE** (*n* = 60)	**TAU** (*n* = 56)
**Demographic information**		
Age, mean (SD)	41 (10)	44 (9)
Gender (female), n (%)	33 (55)	36 (64)
Education, mean (SD)	16 (2)	16 (3)
Marital status, n (%)		
Married/co-habitant	43 (72)	34 (61)
Divorced/separated/single	17 (28)	22 (39)
**Clinical information**		
Cause of injury, n (%) (*n* = 115)		
Fall	19 (32)	30 (54)
Transport	12 (20.5)	11 (20)
Other	28 (47.5)	15 (26)
GCS, median (range) (*n* = 114)	15 (10–15)	15 (11–15)
LOC, n (%), (*n* = 115)		
None	31 (51.5)	30 (54.5)
< 30 min	21 (35)	16 (29)
< 24 h	1 (2)	2 (4)
Not registered	7 (11.5)	7 (12.5)
PTA, n (%), (*n* = 115)		
None	25 (42)	26 (47)
< 1 h / < 24 h	25 (41.5)	26 (56)
< 7 days	0 (0)	2 (4)
Not registered	10 (16.5)	1 (2)
Trauma-related CT/MRI findings, n (%)		
Yes	11 (18)	16 (29)
No	45 (75)	35 (62)
No CT/MRI	4 (7)	5 (9)
AIS head score, n (%)		
Minor	34 (57)	25 (44.5)
Moderate	18 (30)	16 (28.5)
Serious / Severe	8 (13)	15 (27)
Extracranial injuries (yes), n (%)	28 (47)	25 (45)
**Work factors**		
Occupation type (white collar), n (%)	53 (88)	50 (89)
Occupation category, n (%)		
Military/Academic professions	30 (50)	28 (50)
Leaders	15 (25)	13 (23)
Office/Sales	10 (17)	9 (16)
Craft/Machine	5 (8)	6 (11)
Operators/Transportation/Cleaning		
Employment duration (months), median (IQR), (*n* = 114)	54 (114)	42 (108)
Full time position (yes), n (%)	55 (92)	48 (86)
Enterprise size, n (%)		
< 250 employees	33 (55)	40 (71.5)
> 250 employees	27 (45)	16 (28.5)
Sick listed, *n* (%) 80–100% 50–79%	48 (80) 12 (20)	46 (82) 10 (18)
**EQ-5D-5L**		
Index value, mean (SD)	0.648 (0.152)	0.713 (0.116)

*Notes*: *CCT* Compensatory Cognitive Training, *TAU *treatment as usual, *SD* standard deviation, *GCS* Glasgow Coma Scale, *LOC* loss of consciousness, *PTA* post-traumatic amnesia, *AIS* Abbreviated Injury Scale, *IQR* interquartile range

### Study interventions

CCT is a manualised intervention targeting post-concussive symptom management and cognitive symptoms [15, 16]. The intervention was provided by a clinical psychologist and a MD in groups of two to five participants for 2 h weekly over 10 weeks. Each session covered the topics through a combination of psychoeducation and compensatory strategy training. The vocational part of the intervention was based on elements from the Individual Placement and Support model of SE [12, 17]. Follow-up was customised to the individual participants’ needs and entailed mapping resources, limitations and work tasks, followed by guidance, advice and work task accommodations. The SE intervention was provided by three employment specialists for a total of 6 months. The CCT and SE interventions were provided in parallel and therapists providing the interventions collaborated closely to ensure implementation of strategies and compensatory techniques at the workplace.

TAU consisted of assessment and treatment provided by a multidisciplinary team (physiatrist, neuropsychologist, physiotherapist, occupational therapist and social worker) at a specialized TBI outpatient clinic at the Dept. Of Physical Medicine and Rehabilitation, OUH. Follow-up consisted of individual contacts and an education group focused on nonspecific education about TBI and discussion of common problems in daily life following TBI. The education group consisted of four weekly meetings of 2 h, each led by a different professional. TAU was provided for a maximum of 6 months.

### Costs of the study intervention and TAU

The costs of the treatment provided in the intervention and control groups were calculated from each therapist’s gross annual salary, while also considering social costs (employers’ costs for mandatory and supplementary pension plans and insurances, factor of 1.4 in Norway). Costs of the CCT intervention and educational group provided in TAU were further adjusted for mean number of participants in each group (3 in CCT and 7 in educational group). The unit costs and frequency of treatment provided in CCT-SE and TAU are presented in Additional file 3.

### Costs of healthcare, informal care and production loss

Information regarding the use of healthcare services, informal care and sickness absence was collected at 3, 6 and 12 months after study inclusion. The information was self-reported by the participants, including only trauma-related treatment and follow-up, using a questionnaire specifically designed for that purpose (developed by EAA). The questionnaire included number of visits to a general practitioner, physical therapist, chiropractor, contract specialists (dentist, neurologist, ophthalmologist, orthoptist/optician, otorhinolaryngologist and psychologist) and out-of-pocket services (naprapath and osteopath). Information about informal care was collected by asking participants if they had received informal care by friends or family since last follow-up and, if so, number of hours receiving assistance per week. We also recorded information about gross annual salary and productivity loss, which included work status in terms of sickness absence (percentage and duration) at each follow-up. We did not assess the use of medications, distance and transportation related to the healthcare utilisation and, hence, could not calculate medication and transportation costs. The cost of productivity loss was based on the participant’s gross annual salary, and was calculated by subtracting weekly wage for full time work (i.e., pre-injury level of work) from weekly wage for actual number of hours worked per week at each follow-up (i.e., taking into account sickness absence). Costs of informal care was calculated using the opportunity cost method (i.e. time spend providing informal care was valued as paid working time [18], multiplying the mean hourly wage in Norway [19] by number of hours of informal care per week. Health service utilisation and related costs, including formal primary and secondary care reimbursed by the government, were categorized according to the CCT-SE and TAU groups. Total care costs also included informal care and out-of-pocket services (i.e. services not reimbursed by the government). Total societal costs comprised total care costs in addition to costs related to productivity loss. Cost categories and unit costs are presented in Table 2. The majority of unit costs were based on the reimbursement schemes. Costs were estimated on a present-value basis of Euro (€) in 2019 (€1 = Norwegian Kroner [NOK] 10).

**Table 2:** Cost categories, units, valuation and unite price in Euro

**Service**	**Unit**	**Unit cost (**€**)**	**Source**
**Primary care**			
General practitioner	Per visit	50	NOMA, 2019-2020, general practitioner consultation
Physiotherapist, assessment	1 h	92	The Norwegian Physiotherapy Association, 2019
Physiotherapist, treatment	30 min	62	The Norwegian Physiotherapy Association, 2019
Psychologist, assessment	1 h	141	HELFO, 2020
Psychologist, treatment	1 h	110	HELFO, 2020
Chiropractor, assessment	Per visit	30	HELFO, 2020
Chiropractor, treatment	Per visit	14	HELFO, 2020
**Contract specialists**			
Neurology	Per assessment	131	NOMA, 2019-2020, specialist health service consultation
Dentistry	Per assessment	159	HELFO, 2020
Ophthalmology	Per assessment	101	NOMA, 2019–2020, specialist health service consultation
Otorhinolaryngology	Per assessment	94	NOMA, 2019–2020, specialist health service consultation
Optometry/orthoptics	Per assessment	94	NOMA, 2019–2020, specialist health service consultation
**Informal care**			
	Per hour	32	Statistics Norway, 2019
Production loss*			
Gross wage	Per hour	35	Self-reported income
**Out-of-pocket**			
Naprapathy	Per visit	70	Estimate
Osteopathy	Per visit	90	Estimate

*Notes*: *NOMA* Norwegian Medicines Agency, *HELFO* The Norwegian Health Economics Administration

### Health outcome

The primary cost-effectiveness analysis was based on the difference between CCT-SE and TAU in number of days until return to pre-injury work level (i.e., cost per day earlier back to work). As the secondary outcome, cost-effectiveness was evaluated in terms of quality-adjusted life-years, QALYs, where 1 QALY is equal to 1 year in full health. This was measured using utility scores derived from the 5-level EQ-5D version (EQ-5D-5L) based on five dimensions: mobility, self-care, usual activities, pain/discomfort and anxiety/depression [20]. EQ-5D-5L was administered at baseline and follow-ups (3, 6 and 12 months), and valued using the Danish tariff as a Norwegian tariff is currently not available [21].

### Statistical analysis

Descriptive statistics were used to present participants’ characteristics, costs of the CCT-SE intervention and TAU, health- and informal care and productivity loss, with mean and standard deviation (SD) or median and inter-quartile range (IQR) for continuous variables, and proportions and percentages or range for categorical variables.

In the cost-effectiveness analysis, the differences in outcomes of CCT-SE and TAU were compared to difference in costs.

The primary cost-effectiveness analysis was defined by the incremental cost-effectiveness ratio (ICER), given by

1 $$ICER=\frac{{Costs}_{CCT-SE}-{Costs}_{TAU}}{\left({\#days back to work}_{CCT-SE}-{\#days back to work}_{TAU}\right)*(-1)}=\frac{incremental costs}{\#days earlier back to work}$$

where the nominator in Eq. (1) is the incremental cost, while the denominator in Eq. (1) can be interpreted as number of days earlier back to pre-injury work level in the CCT-SE group. The costs included in the primary analysis were total healthcare costs (i.e., costs of the CCT-SE intervention or TAU and other healthcare services).

The secondary cost-effectiveness analysis was based on QALYs as the health outcome. There were 15 participants with missing EQ-5D-5L values. The two missing values in the TAU group at baseline were replaced by the average value for the TAU group. When a participants had a missing observation between two observation points on the time line, we assumed the relationship between the adjacent recorded observations to be linear. For instance, for missing values at 3 months we assumed a linear trend from the observations at baseline to 3 months was carried onwards to 6 months. Further, for participants with reported EQ-5D-5L at baseline and 3 months, but not at 6 and 12 months, we assumed that the trend from baseline to 3 months were carried onwards to 6 months, which resulted in two additional observations being replaced. For missing values at 12 months, we assumed the same EQ-5D-5L value as observation at 6 months, which resulted in 11 observations being replaced.

We estimated the QALYs for each individual by area under the curve (AUC), where we used the EQ-5D-5L values at baseline, 3, 6 and 12 months follow-ups and assumed linear changes in HRQoL over time [22]. From Table 1 we observed that the EQ-5D-5L baseline values were significantly different between CCT-SE and TAU (*p* = 0.013). To adjust for the difference, we applied two methods. Firstly, based on the method presented by Manca et al. [22], we used regression analysis to adjust estimated QALYs for EQ-5D-5L value at baseline and treatment group (see Additional file 4). Secondly, we parallel shifted the observation for the intervention group upwards and equal to the difference at baseline, equal to 0.065. Analysis based on the raw data are presented in Additional file 5. The costs included both total healthcare and societal costs. Lastly, we have included an analysis comparing the improvement in HRQoL values from baseline to 12 months. In this alternative, the incremental effect will be interpreted as the difference in incremental improvement on the HRQoL value over the 12 months follow-up period.

For the secondary cost-effectiveness analysis where QALYs were the main health outcome, we estimated the net monetary benefit (NMB), which is defined by

2 $$NMB=Incremental QALYs* \lambda -Incremental costs$$

where λ refers to the threshold value for a QALY gained. If the NMB is lower or equal to zero, the intervention is considered cost-effective. According to Norwegian guidelines the threshold value depends on the severity of the condition defined by the absolute shortfall (number of lost years in good health), and varies from about € 27 500 to € 1 000 00 [23]. In the estimation of NMB, we applied € 27 500 as the threshold value.

To estimate cost-effectiveness with the change in HRQoL from baseline to 12 months, the results were presented by ICER, defined by Eq. (1) with differences in change in HRQoL.

To account for uncertainty in the outcomes (number of days back to work, QALYs, change in HRQoL and costs) between individuals, we conducted a sensitivity analysis by the bootstrap methods with replacement including 1000 iterations. The results of the 1000 iterations of mean ICERs are presented in a cost-effectiveness plane, and for the secondary cost-effectiveness analysis the cost-effectiveness acceptability curves (CEACs) were presented, reporting the likelihood of the CCT-SE intervention to be cost-effective (number of simulations falling below the threshold) according to different threshold values.

## Results

The costs of the CCT-SE and TAU interventions are provided in Table 3. The results revealed a total cost difference of approximately € 550 (*p* < 0.001) where the TAU treatment was associated with significantly lower costs than the CCT-SE intervention.

**Table 3:** Costs of the CCT-SE and TAU interventions

Treatment	Mean (95% CI)
**CCT-SE** (*n* = 58)	
CCT	1138 (1122–1155)
SE	237 (192–281)
**Total CCT-SE**	**1375 (1331–1419)**
**TAU** (*n* = 55)	
Physiatrist	284 (245–322)
Neuropsychologist	79 (22–136)
Physiotherapist	56 (37–74)
Occupational therapist	137 (111–129)
Social worker	55 (32–78)
Educational group	210 (169–250)
**Total TAU**	**820 (691–950)**

*Notes*: *CI* confidence interval, *CCT *Compensatory Cognitive Training, *SE *supported employment, *TAU *treatment as usual

The total costs of primary and secondary healthcare services, out-of-pocket services, informal care and productivity loss over the 12-month follow-up period are provided in Table 4 (see Additional file 6 for detailed information on costs at each follow-up time-point). Costs were mainly related to productivity loss, accounting for 84% of total costs. Costs of healthcare services and informal care each accounted for 8% of total costs including intervention costs. See Additional file 7 for frequency of primary and secondary healthcare service utilization and hours of informal care. The mean cost of healthcare services (sum of intervention costs, primary care, contract specialists and other) was higher in the CCT-SE group (€ 3281) compared to TAU (€ 2300). The most frequently used healthcare services in both CCT-SE and TAU was general practitioner (median [range] number og visits in the CCT-SE group was 8 [3-19] and 9 [1-19] in the TAU group). However, the costliest service in both groups was physical therapy with a mean (SD) of € 756 (1090) for participants in CCT-SE and € 516 (1116) for participants in TAU. The total mean costs of informal care and productivity loss was lower in the CCT-SE group compared to TAU.

**Table 4:** Total costs of primary healthcare services, contract specialists, other healthcare services, informal care and production loss by treatment group

	**CCT-SE** (*n* = 58) mean (SD)	**TAU** (*n* = 55) mean (SD)	Difference	*p*-value
**Primary care**				
General practitioner	415 (202)	447 (234)	-32	.432
Physiotherapist	756 (1090)	516 (1116)	240	.244
Chiropractor	96 (177)	40 (115)	56	.047
**Contract specialists**				
Dentist	61 (414)	3 (21)	58	.297
Neurologist	37 (113)	7 (39)	30	.062
Opthalmologist	13 (47)	32 (86)	-19	.141
Orthoptist	6 (38)	2 (13)	4	.393
Otorhinolaryngologist	13 (41)	10 (34)	3	.726
Psychologist	302 (772)	278 (729)	24	.866
**Other**				
Naprapathy	17 (98)	12 (50)	5	.764
Osteopathy	61 (204)	21 (145)	40	.222
Optician	118 (191)	111 (166)	7	.822
**Informal care**				
	2761 (5096)	3591 (7325)	-830	.496
**Production loss**				
	30,738 (19,244)	33,401 (19,621)	-2663	.495

*Notes*: *CCT *Compensatory Cognitive Training, *SE *supported employment, *TAU *treatment as usual, *SD *standard deviation

### Cost-effectiveness

Three different cost-effectiveness analyses were conducted. The primary analysis was based on the number of days until return to pre-injury work levels. From Table 5, we see that from a healthcare perspective, the ICER was € 56 per day earlier back to work (897/16) for the CCT-SE group compared to TAU. The mean production loss per day for the CCT-SE group was € 275, thus outweighing the cost per day earlier back to work.

**Table 5:** Health outcomes, costs, incremental values, ICER and NMB according to health outcome and cost perspective. Numbers are based on bootstrapped analyses and reported in Euro

**Alternative analyses (*n* = CCT-SE/TAU)**	**CCT-SE**	**TAU**	**Incremental** **values *(95% CI)***	**ICER**	**NMB**
**Primary analysis:**					
Healthcare costs (*n* = 56/55)	2,770	1,872	897 (-109–1,565)		
# of days earlier RTW	345	360	16 *(-68 – 90)*	56	
**Secondary analysis:**					
Healthcare costs (*n* = 56/55)	3,006	2,025	979 *(-159 – 1,877)*		
Societal costs (*n* = 46/44)	36,296	37,615	-1,319 *(-19,643 – 11,807)*		
QALYs—adjusted for baseline					
Healthcare			0.009 (0.018)*		-561
Societal					1,566
QALYs—parallell adjusted					
Healthcare	0.785	0.754	0.031 *(-0.043 – 0.102)*		-132
Societal	0.809	0.771	0.037 *(-0.037–0.121)*		2,336
HRQoL – improvement					
Healthcare	0.111	0.071	0.042 *(-0.036–0.138)*	19,260	
Societal	0.107	0.096	0.012 *(-0.085–0.088)*	-109,900**	

^*^St.error and incremental effect independent of perspective. ^**^ICER with a positive incremental health gain and negative (cost saving) incremental costs

The second and third analyses were based on the secondary outcome, QALYs. When estimating the incremental QALYs by adjusting for EQ-5D-5L values at baseline, the incremental QALYs was 0.009 (see the regression output reported in Additional file 4 ). Given a threshold value of € 27,500, the NMB from the healthcare perspective was € -561 (0.009*27,500–979), indicating that CCT-SE was not a cost-effective alternative to TAU at the given threshold. From the societal perspective, the NMB was € 1,566 (0.009*27,500-(-1,319)), where the incremental costs are negative indicating CCT-SE to be a cost saving alternative compared to TAU.

**Fig. 1:**
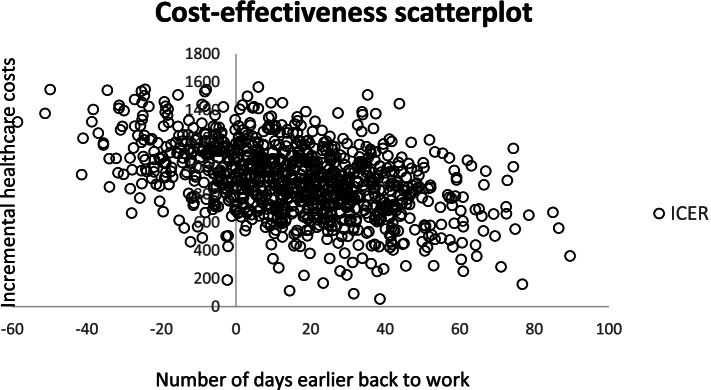
Scatterplot of 1000 bootstrapped iteration of health care costs and number of days earlier back to work as health outcome

**Fig. 2:**
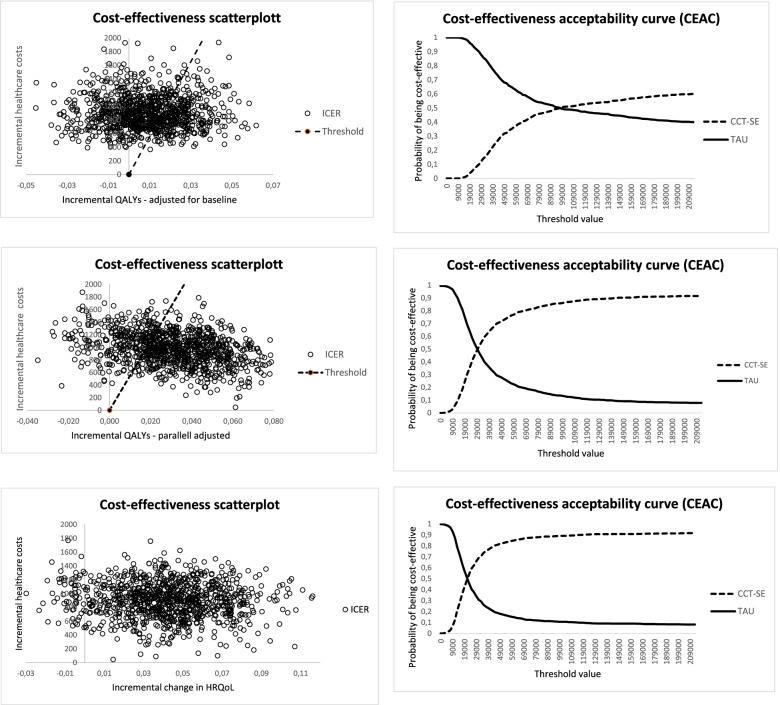
Scatterplots and CEACs of 1000 bootstrapped iteration of incremental health care costs. For each row there are different assumptions with regard to the estimation of health outcomes. In the upper row, we have estimated incremental QALY by adjusting for baseline EQ-5D-5L values. In the second row all EQ-5D-5L values for all observation points in the CCT-SE has been adjusted upwards with the difference in EQ-5D-5L between CCT-SE and TAU at baseline, while in the third row we have estimated the change in HRQoL value from baseline to 12 months

In the next alternative, where we parallel adjusted the EQ-5D-5L values equal to the difference at baseline, the NMB was € -132 (0.031*27,500–979) and € 2,336 (0.037*27,500-(-1,319)) from a healthcare and societal perspective, respectively. Lastly, when the health outcome was measured as a change in HRQoL from baseline to 12 months, the ICER was € 19,260 per QALY gained (979/0.042), and € – 109,900 per QALY gained (-1,319/0.012) for health care and societal costs, respectively. In Fig. 1, we have presented the uncertainty analysis for the primary outcome (number of days earlier return to work). All scatters represent estimated mean incremental health care costs and number of days earlier back to work. Scatters above the horizontal axes indicate that CCT-SE is more costly than TAU (99.9% of the scatters), while scatters to the right of the y-axes indicate a positive effect of CCT-SE on number of days earlier back to work (76% of the scatters). In Fig. 2, scatterplots and CEACs are presented for three health outcomes. The upper two figures present the results from the analysis where the QALYs gained was adjusted for the difference in baseline EQ-5D-5L value. All scatters are above the horizontal axes, indicating that CCT-SE is more costly than TAU when only health care costs are included. From the CEAC we see that the probability of CCT-SE being cost-effective is higher than TAU for threshold values above € 98,000.

**Fig. 3:**
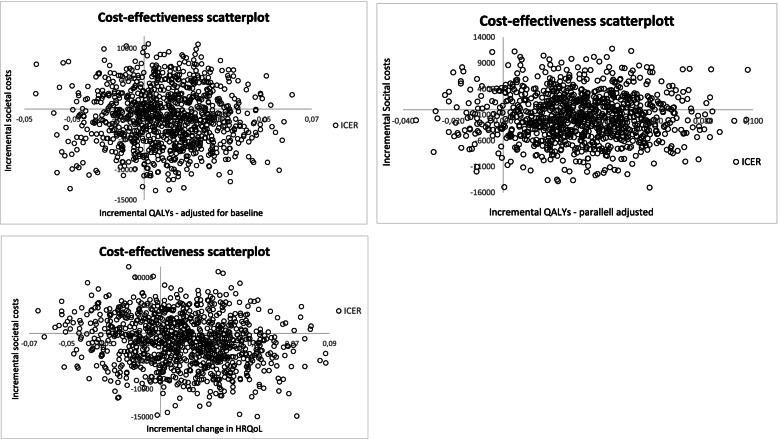
Scatterplot of 1000 bootstrapped iteration of incremental societal costs and two different methods for estimation of QALYs. In the left plot incremental QALYs have been adjusted for the EQ-5D-5L measures at baseline, while in the right plot all values in the CCT-SE have been adjusted upwards with the difference in EQ-5D-5L between CCT-SE and TAU at baseline

The two figures in the middle present the results based on a parallel shift of the EQ-5D-5L values equal to the difference at baseline. From the scatterplot we see that 92% of the iterations have a positive incremental QALY. The CEAC illustrates that at a threshold of € 32,000, the likelihood for CCT-SE and TAU to be cost-effective is equal to 50%. For threshold values above € 32,000, the likelihood for CCT-SE to be cost-effective is higher than TAU reaching 80% at values above € 69,000. In the last row we have reported results based on difference in the improvement of HRQoL scores from baseline to 12 months between CCT-SE and TAU. The costs are the same as for the two other alternatives, while 94% of the scatters indicate a positive effect of CCT-SE on the change in HRQoL over 12 months. For threshold values above € 22,000, CCT-SE is more likely to be cost-effective compared to TAU. In Fig. 3, the above analyses were re-run, but included societal costs. We see that the proportion of iterations with a negative incremental value (indicating CCT-SE to be cost-saving) was equal to about 63%.

## Discussion

The main goal of this study was to describe the costs of two rehabilitation interventions and determine if a combined cognitive and vocational rehabilitation intervention was preferable in terms of costs, effect and utility compared to multidisciplinary TAU provided in the specialized healthcare service. We considered both health and societal perspectives, including costs of healthcare services, informal care and productivity loss. We were unable to find evidence to support the hypothesis that CCT-SE was a cost-effective alternative from the healthcare perspective. However, we found a positive effect of the CCT-SE intervention on change in HRQoL over 12 months. Furthermore, from a societal perspective, considering costs related to productivity loss and informal care, the CCT-SE intervention was a cost-effective alternative to TAU.

### Healthcare and societal costs

Considering both healthcare and societal costs, costs associated with healthcare services were relatively low and accounted for 8% of the total societal costs. This could be related to the injury severity levels of the participants included in this study; patients with mild and moderate TBI are less likely in need of complex and expensive healthcare services compared to those with severe TBI [24]. However, at the population level, costs following mild TBI far exceed those of more severe injuries as a result of the high incidence of mild TBI [25, 26]. Moreover, indirect costs related to loss of productivity are generally considered to exceed the direct costs associated with healthcare utilization after TBI [27].

Healthcare costs were higher in the CCT-SE group which may in part be due to the small number of patients in some of the CCT groups leading to use of more healthcare resources per patient and consequently higher healthcare costs. The intervention was provided as part of a research project operating under resource and time restrictions, while providing the intervention in a clinical setting could allow for larger group sizes as the intervention developer notes that CCT may be delivered in groups of up to 7 participants [15]. The baseline EQ-5D-5L index values were significantly lower in the CCT-SE group compared to TAU despite randomisation. Lower initial HRQoL could indicate a greater need for healthcare services, thus might have contributed to the higher consumption and costs of healthcare services observed in the CCT-SE group. However, these findings may also indicate that the CCT-SE intervention did not cover the participants specific needs for healthcare services such as treatment of somatic problems (headache, dizziness, nausea, vision problems) leading them to seek more additional services.

Costs related to productivity loss constituted the largest costs in both groups, accounting for 84% of total costs. These costs were lower in the intervention group supporting the main findings of the RCT, showing a significant difference in work participation between CCT-SE and TAU at 3 months follow-up. Additionally, participants in the CCT-SE group returned to preinjury work levels earlier which might indicate a beneficial effect of the combined cognitive and vocational intervention [10].

A substantial proportion of support required to live at home after TBI is provided by informal caregivers, family members and friends, but these costs are seldom reported in clinical trials. In this study, informal care costs accounted for 8% of the total costs, and were lower in the CCT-SE group compared to TAU. The CCT intervention provides psychoeducation about TBI and strategies to manage cognitive and other symptoms which may have had a positive influence on independence in functioning. An alternative explanation may be found in differences in the demographic characteristics of the participants. Compared to CCT-SE, a higher proportion of participants in the TAU group (22% vs 17%) reported that they were single and thus may have needed more support from their environment in managing the consequences of their TBI.

### Cost-effectiveness

This is the first study to evaluate the cost-effectiveness of a combined cognitive and vocational rehabilitation intervention in patients with mild and moderate TBI. Overall, the findings show that the incremental health care costs are positive, while the incremental societal costs are negative, which implies that CCT-SE intervention is a cost-saving alternative to TAU from the societal perspective. The incremental effect depends on the analysis. In the primary analysis, the CCT-SE intervention resulted in earlier return to work. In the secondary analysis, when measuring the health outcome in QALYs, the size of the incremental effect depended on whether and how the imbalance in HRQoL at inclusion were adjusted. Both groups improved their HRQoL, and improvements over 12 months were in line with previously reported estimates of minimally clinical important difference in EQ-5D index values ranging from 0.03 to 0.52 [28]. The change in HRQoL was, however, greater in the CCT-SE group, suggesting a positive effect of intervention.

In 2016, the suggested threshold value for a QALY gained in Norway was € 27 500 – € 100 000 per QALY for the lowest to severest grade patients [23]. In this study, when we parallel adjusted the EQ-5D-5L values equal to the difference at baseline, the cost-effectiveness of CCT-SE and TAU was equal at the threshold of € 32 000. However, for threshold values above € 32 000, the likelihood for CCT-SE to be cost-effective was higher than TAU. Further, when considering the between-group incremental difference in HRQoL from baseline to 12 months, CCT-SE is more likely to be cost-effective compared to TAU from threshold values above € 22 000. Mild TBI is a highly prevalent condition with a high impact on productivity loss and societal costs and could in this context be considered as a more severe condition. In this study, costs associated with productivity loss accounted for 84% of the total costs, highlighting the impact of mild TBI on societal costs.

It is difficult to compare results from this study with previous studies due to substantial variation regarding methodology, patients’ clinical characteristics and intervention content. The majority of studies assessing the cost-effectiveness of rehabilitation after TBI are carried out on severe TBI [29–31], with results supporting the cost-effectiveness of early transfer to a dedicated neurocritical care unit or specialized rehabilitation. Our intervention can also be considered as an early, specialized intervention launched 8–12 weeks after mild-to-moderate TBI. Thus, the study findings give further support to the notion that targeted early intervention programs for patients with TBI might be cost-effective, in this case due to the costs associated with informal care and productivity loss.

### Strengths

This study systematically evaluated and compared the costs related to an intervention program and standard care both during and after the intervention phase for working age patients with mild-to-moderate TBI. The cost evaluation did not only include costs related to the study treatments and healthcare services, but also productivity loss and informal care from family caregivers related to daily life consequences of the injury, reflecting meaningful impacts of TBI on families and society. The societal costs were dominating both with regard to the total costs as well as the incremental costs, which resulted in CCT-SE being a cost-saving alternative. Guidelines for economic evaluations in several countries recommend analysis to be conducted from a health care perspective [32]. This study has illustrated that for some patient groups and types of interventions, a health care perspective does not fully represent the consequences an intervention could imply.

### Limitations

There was a significant difference in baseline EQ-5D index values despite following randomisation procedures. The lower baseline index value in the CCT-SE group may have allowed for greater improvement in HRQoL over time compared to TAU and should be considered when interpreting results from this study. There were 28 missing observation points for EQ-5D-5L amounting to approximately 6% of the total number of observations. By manual imputation, we reduced the number of missing information to 11 EQ-5D-5L values (2.4%), which resulted in 56 and 55 observations of QALYs in the CCT-SE and TAU group, respectively. Alternatively we could have applied multiple imputation, but as the number of observations was relatively small, we do not believe that it would change the results. There were also missing information in the estimation of production loss and informal care, resulting in a reduction in number of observations to 46 and 44 for CCT-SE and TAU, respectively. Replacing production loss and informal care, was not possible based on the information in the study. In situations where missing values are not at random, the results in the cost-effectiveness analysis could be biased. The number of missing values in the analysis applying the health care perspective is relatively small, hence it is likely that the findings are representative. With regard to analysis from the societal perspective, future research should focus on ensuring data collection that reduce the number of missing information to validate the results from our study. The study was conducted within the specialist healthcare service in Norway, where the study intervention was compared with TAU which is a standard follow-up practice in the study hospital, but may not be representative of practice in other hospitals in Norway or internationally. Thus, the results may not be generalizable to other health care systems with different care practices or economic conditions.

The cost of informal care was valued in terms of mean hourly wage multiplied by hours spend on providing care (i.e. productivity loss of the caregiver). However, we did not register whether the informal care was provided during work hours or leisure time, thus time spent providing care did not necessarily equal lost productivity for the informal caregivers. Moreover, hours of informal care per week was reported by the participants which may have led to an over- or under-estimation. The operationalization and valuation of informal care is a subject of discussion [18]. While some argue that valuing informal care as lost wage may lead to an over-estimation of costs, others argue that leisure time should be valued equal or above market wage rate. We applied the human capital approach [33] in the analyses, assuming that a person on sick leave cannot be replaced and hence all absence from work is included as a cost. Alternatively, we could have applied the friction cost approach, where only the costs related to replacing a person on sick leave is accounted for in the cost [33]. This approach would most likely have resulted in lower societal costs. Obtaining sick-leave and healthcare claims for the participants would provide a more valid measure of healthcare use and cost; however, the self-reported utilization is necessary when including costs for non-covered services such as informal care and out-of pocket healthcare services. Moreover, self-reported productivity loss and healthcare utilization could be considered a limitation if respondents report false values or have difficulty remembering. However, the study participants had sustained mild-to-moderate TBIs, and their knowledge of personal work-related data was not suspected to be notably affected. In addition, neuropsychological assessment at baseline showed that severe memory or attention problems were not present to such a degree that one would expect invalid reports [34].

## Conclusion

Compared to TAU, CCT-SE was associated with higher healthcare costs, but lower costs related to informal care and productivity loss. Costs associated with productivity loss accounted for the majority of the total costs, further supporting the implementation of effective intervention programs targeted at return-to-work for this population. The cost-effectiveness of the CCT-SE intervention differed according to a healthcare or societal perspective. The CCT-SE intervention was not cost-effective from a healthcare perspective. However, when also considering productivity loss and informal care, CCT-SE was a cost-effective alternative to TAU.

## Supplementary Information

**Additional file 1.** Flow chart of participant recruitment.

**Additional file 2. **Proportion of responses by level of severity for EQ-5D-5L dimensions at baseline by treatment group (CCT-SE, Compensatory cognitive training and supported employemnt and TAU, treatment as usual).

**Additional file 3.** Unit costs and frequency of CCT-SE and TAU.

**Additional file 4. **Health outcome and regression for adjustment at baseline.

**Additional file 5. **Analysis based on the raw data.

**Additional file 6.** Costs by treatment group from baseline to 3 months, 3-6 months and 6-12-months follow-up.

**Additional file 7.** Frequency of healthcare service use, out-of-pocket services and informal care by treatment group at 3-, 6- and 12-months follow-up and in total.

## Data Availability

The dataset used and analysed during the current study are available from the corresponding author on reasonable request.

## Abbreviations

AUC: Area under the curve
CCT: Compensatory cognitive training
CEAC: Cost-effectiveness acceptability curve
ICER: Incremental cost-effectiveness ratio
NMB: Net monetary benefit
NOK: Norwegian kroner
OUH: Oslo university hospital
QALY: Quality adjusted life year
RCT: Randomised controlled trial
RTW: Return to work
SE: Supported employment
TAU: Treatment as usual
TBI: Traumatic brain injury
