# Supplementary material for: Cost-effectiveness analysis of combined cognitive and vocational rehabilitation in patients with mild-to-moderate TBI: results from a randomized controlled trial
Source: BMC Health Serv Res. 2022 Feb 12;22:185. doi: 10.1186/s12913-022-07585-3 (PMC8840547; doi:10.1186/s12913-022-07585-3)
Supplement: Supplementary file 1 — Additional file 1. Flow chart of participant recruitment. [file 12913_2022_7585_MOESM1_ESM.docx]

**Additional file 1.** Flow chart of participant recruitment.

Excluded (n = 476)

- Not meeting inclusion criteria (n = 437)

Time since injury (n = 138)

Age (n = 81)

Sick leave % < 50% (n = 50)

Disability pension/not working (n = 41)

Student (n = 32)

Other reasons (n = 95)

- Declined to participate (n = 39)

Randomised (n = 116)

## Allocation

## Analysis

Analysed (n = 56)

Analysed (n = 60)

## Follow-up 3 months

## Follow-up 6 months

## Follow-up 12 months

Lost to follow-up (n = 5)

Lost to follow-up (n = 4)

Lost to follow-up (n = 1)

Lost to follow-up) (n = 2)

Lost to follow-up (n = 0)

Lost to follow-up (n = 0)

Assessed for eligibility (n = 592)

Allocated to CCT-SE (n = 60)

♦ Received allocated intervention (n = 58)

♦ Did not receive allocated intervention (drop-out) (n = 2)

Allocated to TAU (n = 56)

♦ Received allocated intervention (n = 55)

♦ Did not receive allocated intervention (drop-out) (n = 1)
