# Supplementary material for: Cost-effectiveness analysis of combined cognitive and vocational rehabilitation in patients with mild-to-moderate TBI: results from a randomized controlled trial
Source: BMC Health Serv Res. 2022 Feb 12;22:185. doi: 10.1186/s12913-022-07585-3 (PMC8840547; doi:10.1186/s12913-022-07585-3)
Supplement: Supplementary file 2 — Additional file 2. Proportion of responses by level of severity for EQ-5D-5L dimensions at baseline by treatment group (CCT-SE, Compensatory cognitive training and supported employemnt and TAU, treatment as usual). [file 12913_2022_7585_MOESM2_ESM.docx]

**Additional file 2.** Proportion of responses by level of severity for EQ-5D-5L dimensions at baseline by treatment group (CCT-SE, Compensatory cognitive training and supported employemnt and TAU, treatment as usual).
