# Supplementary material for: Cost-effectiveness analysis of combined cognitive and vocational rehabilitation in patients with mild-to-moderate TBI: results from a randomized controlled trial
Source: BMC Health Serv Res. 2022 Feb 12;22:185. doi: 10.1186/s12913-022-07585-3 (PMC8840547; doi:10.1186/s12913-022-07585-3)
Supplement: Supplementary file 3 — Additional file 3. Unit costs and frequency of CCT-SE and TAU. [file 12913_2022_7585_MOESM3_ESM.docx]

**Additional file 3.** Unit costs and frequency of CCT-SE and TAU.

| **Treatment group** | **Unit** | **Unit cost (**€**)** | **Frequency**  **Mean (SD) Median (range)** | |
| --- | --- | --- | --- | --- |
| **CCT-SE** | | | | |
| Compensatory Cognitive Training | Per session (2 h) | 115 | 10 (1) | 10 |
| Supported employment | Per contact | 80 | 3 (2) | 2.5 (0-11) |
| Total |  |  | 13 (3) | 12.5 (10-21) |
| **TAU** | | | | |
| Physiatrist | Per visit | 123 | 2 (1) | 2 (1-8) |
| Neuropsychologist | Per visit | 108 | 1 (2) | 0 (0-14) |
| Physiotherapist | Per visit | 71 | 1 (1) | 1 (0-4) |
| Occupational therapist | Per visit | 72 | 2 (1) | 2 (0-7) |
| Social worker | Per visit | 72 | 1 (1) | 0 (0-4) |
| Educational group | Per session (2 h) | 84 | 3 (2) | 3 (0-6) |
| Total |  |  | 9 (5) | 9 (1-28) |

*Notes*: SD, standard deviation; CCT, Compensatory Cognitive Training; TAU, treatment as usual.
