# Supplementary material for: Cost-effectiveness analysis of combined cognitive and vocational rehabilitation in patients with mild-to-moderate TBI: results from a randomized controlled trial
Source: BMC Health Serv Res. 2022 Feb 12;22:185. doi: 10.1186/s12913-022-07585-3 (PMC8840547; doi:10.1186/s12913-022-07585-3)
Supplement: Supplementary file 4 — Additional file 4. Health outcome and regression for adjustment at baseline. [file 12913_2022_7585_MOESM4_ESM.docx]

**Additional file 4**. Health outcome and regression for adjustment at baseline.

**Health outcome**

The table below shows unadjusted and adjusted EQ-5D-5L index values at baseline, 3, 6 and 12 months follow up in the CCT-SE and TAU groups. Change from baseline to 12 months in unadjusted data was 0.112 (0.648-0.760) for the intervention group, and 0.071 (0.713-0.784) for the TAU group, implying an incremental difference in HRQoL improvement of 0.041.

Change from baseline to 12 months in adjusted data (with differences at baseline) was 0.118 (0.713-0.831) for the intervention group, and 0.071 (0.713-0.784) for the TAU group, implying an incremental difference in HRQoL improvement of 0.044.

**Table.** Unadjusted and adjusted EQ-5D-5L index values at baseline, 3, 6 and 12 months follow-up.

| **Observation time** | **CCT-SE**  Mean (SD) | **TAU**  Mean (SD) |
| --- | --- | --- |
| **Unadjusted data** |  |  |
| Baseline (n=60/56) | 0.648 (0.152) | 0.713 (0.114) |
| 3 months (n=58/55) | 0.709 (0.133) | 0.748 (0.109) |
| 6 months (n=58/55) | 0.744 (0.151) | 0.753 (0.164) |
| 12 months (n=56/55) | 0.762 (0.133) | 0.784 (0.152) |
| QALYs – unadjusted n=56/55) | 0.723 (0.123) | 0.754 (0.116) |
| **Adjusted data** |  |  |
| Baseline (n=60/56) | 0.713 (0.152) | 0.713 (0.116) |
| 3 months (n=58/55) | 0.773 (0.131) | 0.748 (0.109) |
| 6 months (n=58/55) | 0.803 (0.143) | 0.753 (0.164) |
| 12 months (n=56/55) | 0.819 (0.130) | 0.784 (0.152) |
| QALYs – adjusted (n=56/55) | 0.784 (0.117) | 0.754 (0.119) |

*Notes*: CCT, Compensatory cognitive training; TAU, treatment as usual; SD, standard deviation.
