# Supplementary figures and images for: Cost-effectiveness analysis of combined cognitive and vocational rehabilitation in patients with mild-to-moderate TBI: results from a randomized controlled trial
Source: BMC Health Serv Res. 2022 Feb 12;22:185. doi: 10.1186/s12913-022-07585-3 (PMC8840547; doi:10.1186/s12913-022-07585-3)

| 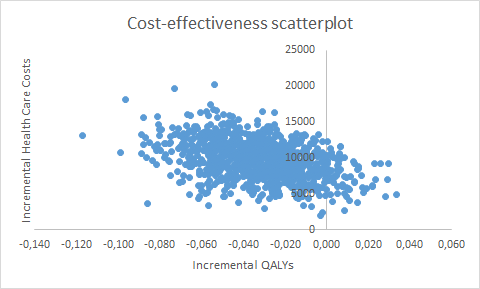 | 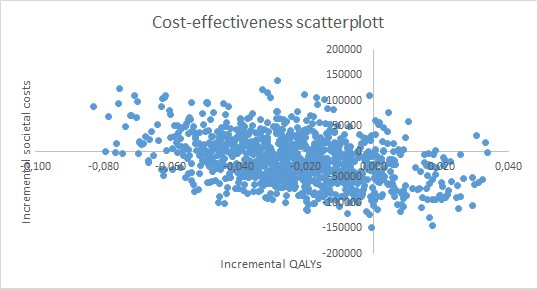 |
| --- | --- |

**Additional file 5**. Analysis based on the raw data.

Supplement: Supplementary file 5 — Additional file 5. Analysis based on the raw data. [file 12913_2022_7585_MOESM5_ESM.docx]
