# Supplementary material for: Cost-effectiveness analysis of combined cognitive and vocational rehabilitation in patients with mild-to-moderate TBI: results from a randomized controlled trial
Source: BMC Health Serv Res. 2022 Feb 12;22:185. doi: 10.1186/s12913-022-07585-3 (PMC8840547; doi:10.1186/s12913-022-07585-3)
Supplement: Supplementary file 6 — Additional file 6. Costs by treatment group from baseline to 3 months, 3-6 months and 6-12-months follow-up. [file 12913_2022_7585_MOESM6_ESM.docx]

| Service | **CCT-SE**  (*n* = 60)  mean (SD) | | | **TAU**  (*n* = 56)  mean (SD) | | |
| --- | --- | --- | --- | --- | --- | --- |
| Follow-up (months) | 0-3 | 3-6 | 6-12 | 0-3 | 3-6 | 6-12 |
| **Primary care** | | | | | | |
| General practitioner | 201 (140) | 137 (79) | 77 (81) | 192 (137) | 144 (111) | 111 (119) |
| Physiotherapist | 214 (367) | 304 (493) | 237 (487) | 134 (202) | 195 (541) | 188 (517) |
| Chiropractor | 28 (67) | 42 (112) | 26 (79) | 10 (34) | 17 (60) | 15 (52) |
| **Contract specialists** | | | | | | |
| Dentist | 53 (410) | 8 (62) | 0 (0) | 3 (21) | 0 (0) | 0 (0) |
| Neurologist | 15 (49) | 11 (50) | 11 (56) | 7 (39) | 0 (0) | 0 (0) |
| Opthalmologist | 5 (22) | 2 (13) | 7 (31) | 13 (9) | 9 (40) | 11 (49) |
| Orthoptist | 5 (27) | 2 (12) | 0 (0) | 2 (13) | 0 (0) | 0 (0) |
| Otorhinolaryngologist | 11 (39) | 2 (12) | 0 (0) | 5 (21) | 0 (0) | 5 (21) |
| Psychologist | 43 (147) | 80 (280) | 179 (558) | 89 (223) | 131 (349) | 58 (250) |
| **Other** | | | | | | |
| Naprapathy | 12 (90) | 0 (0) | 5 (41) | 6 (38) | 6 (33) | 0 (0) |
| Osteopathy | 21 (89) | 18 (139) | 22 (120) | 2 (12) | 19 (144) | 0 (0) |
| Optician | 79 (125) | 27 (69) | 12 (76) | 70 (118) | 29 (64) | 12 (48) |
| **Informal care** | | | | | | |
|  | 888 (1466) | 787 (1503) | 1433 (3200) | 1435 (2382) | 1012 (1917) | 1290 (3740) |
| **Production loss** | | | | | | |
|  | 12198 (6288) | 8609 (5800) | 9930 (9592) | 11986 (6043) | 8993 (5529) | 12422 (10834) |

**Additional file 6**. Costs by treatment group from baseline to 3 months, 3-6 months and 6-12-months follow-up.
