# Supplementary material for: Cost-effectiveness analysis of combined cognitive and vocational rehabilitation in patients with mild-to-moderate TBI: results from a randomized controlled trial
Source: BMC Health Serv Res. 2022 Feb 12;22:185. doi: 10.1186/s12913-022-07585-3 (PMC8840547; doi:10.1186/s12913-022-07585-3)
Supplement: Supplementary file 7 — Additional file 7. Frequency of healthcare service use, out-of-pocket services and informal care by treatment group at 3-, 6- and 12-months follow-up and in total. [file 12913_2022_7585_MOESM7_ESM.docx]

| Service | CCT-SE  (*n* = 60)  median (range) | | | | TAU  (*n* = 56)  median (range) | | | |
| --- | --- | --- | --- | --- | --- | --- | --- | --- |
| Follow-up (months) | 3 | 6 | 12 | Total | 3 | 6 | 12 | Total |
| **Primary care** | | | | | | | | |
| General practitioner | 4 (0-15) | 3 (0-7) | 1 (0-5) | 8 (3-19) | 4 (1-13) | 3 (0-12) | 2 (0-12) | 9 (1-19) |
| Physiotherapist | 0 (0-20) | 0 (0-24) | 0 (0-24) | 1 (0-66) | 1 (0-13) | 0 (0-36) | 0 (0-36) | 2 (0-85) |
| Chiropractor | 0 (0-29) | 0 (0-24) | 0 (0-18) | 0 (0-14) | 0 (0-23) | 0 (0-11) | 0 (0-12) | 0 (0-10) |
| **Contract specialists** | | | | | | | | |
| Dentist | 0 (0-20) | 0 (0-3) | 0 (0) | 0 (0-20) | 0 (0-1) | 0 (0) | 0 (0) | 0 (1) |
| Neurologist | 0 (0-2) | 0 (0-2) | 0 (0-3) | 0 (0-5) | 0 (0-2) | 0 (0) | 0 (0) | 0 (2) |
| Opthalmologist | 0 (0-1) | 0 (0-1) | 0 (0-2) | 0 (0-2) | 0 (0-2) | 0 (0-2) | 0 (0-3) | 0 (0-5) |
| Orthoptist | 0 (0-2) | 0 (0-1) | 0 (0) | 0 (0-3) | 0 (0-1) | 0 (0) | 0 (0) | 0 (0-1) |
| Otorhinolaryngologist | 0 (0-2) | 0 (0-1) | 0 (0) | 0 (0-2) | 0 (0-1) | 0 (0) | 0 (0-2) | 0 (0-2) |
| Psychologist | 0 (0-29) | 0 (0-6) | 0 (0-12) | 0 (0-20) | 0 (0-40) | 0 (0-12) | 0 (0-12) | 0 (0-12) |
| **Other** | | | | | | | | |
| Naprapathy | 0 (0-10) | 0 (0) | 0 (0-4.5) | 0 (0-10) | 0 (0-4) | 0 (0-3) | 0 (0-4.5) | 0 (0-4) |
| Osteopathy | 0 (0-5.5) | 0 (0-12) | 0 (0-10 | 0 (0-12) | 0 (0-1) | 0 (0-12) | 0 (0-10) | 0 (0-12) |
| Optician | 0 (0-4) | 0 (0-3) | 0 (0-6) | 0 (0-11 | 0 (0-4) | 0 (0-3) | 0 (0-3) | 0 (0-7) |
| **Informal care** | | | | | | | | |
| Hours per week | 0 (0-20) | 1 (0-22.5) | 0 (0-18) | 2 (0-50) | 1 (0-30) | 1 (0-30) | 0 (0-30) | 1.5 (0-90) |

**Additional file 7.** Frequency of healthcare service use, out-of-pocket services and informal care by treatment group at 3-, 6- and 12-months follow-up and in total.
